# Pleuroparenchymal fibroelastosis in systemic sclerosis-associated interstitial lung disease

**DOI:** 10.3906/sag-2107-13

**Published:** 2022-01-01

**Authors:** Alper SARI, Ömer ÖNDER, Berkan ARMAĞAN, Ertuğrul Çağrı BÖLEK, Bayram FARİSOĞULLARI, Emre BİLGİN, Gözde Kübra YARDIMCI, Macit ARIYÜREK, Ali AKDOĞAN

**Affiliations:** 1 Department of Rheumatology, Faculty of Medicine, Hacettepe University, Ankara, Turkey; 2 Department of Radiology, Faculty of Medicine, Hacettepe University, Ankara, Turkey

**Keywords:** Systemic sclerosis, interstitial lung disease, pleuroparenchymal fibroelastosis

## Abstract

**Background/aim:**

To explore the frequency and clinical associations of radiologic pleuroparenchymal fibroelastosis (PPFE) in patients with systemic sclerosis-associated interstitial lung disease (SSc-ILD).

**Materials and methods:**

In this single-center retrospective study, high resolution computed tomography (HRCT) images of 105 patients with SSc-ILD were examined for the presence of PPFE. Demographic, clinical, laboratory, and pulmonary function test (PFT) data of patients with and without PPFE were compared.

**Results:**

PPFE was detected in 19 (18.1%) patients (‘definite PPFE’ in 13 and ‘consistent with PPFE’ in 6 patients). Patients with PPFE had higher age and longer disease duration than PPFE (−) patients (p < 0.05 for both). Radiologic usual interstitial pneumoniae (UIP) pattern was more frequent (26.3% vs. 4.7%, p = 0.01) and median force vital capacity (FVC) was lower in patients with PPFE (64% vs. 82%, p = 0.005). Spontaneous pneumothorax developed in one patient with PPFE. More deaths occured in PPFE (+) group during follow-up (31% vs. 11%, p = 0.04).

**Conclusion:**

PPFE on HRCT is not uncommon in SSc-ILD and is associated with radiologic UIP pattern and worse lung functions. Further studies are needed to elucidate the prognostic value of PPFE in SSc-ILD.

## 1. Introduction

Interstitial lung disease (ILD) is a major complication of systemic sclerosis (SSc) and one of the leading causes of mortality and morbidity [1]. Up to 80% of SSc patients had evidence of ILD on high resolution computed tomography (HRCT) however, about %30 of them develop progressive lung fibrosis [2]. Nonspecific interstitial pneumoniae (NSIP) is the most frequent histopathologic and radiologic pattern of SSc-ILD and predominantly involves lower lobes of the lungs [3,4]. Other ILD patterns including usual interstitial pneumoniae (UIP), respiratory bronchiolitis associated ILD and organising pneumoniae are less commonly observed in the course of SSc [4]. Pleuroparenchymal fibroelastosis (PPFE) is a rare disorder characterised by the fibrosis of visceral pleura and the subpleural parenchymal areas of upper lobes [5]. Idiopathic form of PPFE has been listed within the classification of idiopathic interstitial pneumoniaes [6]. On the other hand, PPFE can also occur in association with other conditions including infections, lung or bone marrow transplantation, hypersensitivity pneumonitis, and connective tissue diseases [7]. Little data exists on the frequency and clinical significance of radiological PPFE-like lesions in SSc-ILD [8,9]. This study aims to explore the frequency of PPFE and its association with clinical features in a single-center SSc-ILD cohort.

## 2. Materials and methods

We retrospectively reviewed the charts of patients with SSc seen in outpatient rheumatology department between April 2015 and July 2018. Patients with ILD and available high-resolution computed tomography (HRCT) scan in our institute were identified and included in the study. In all included patients’ classification criteria for SSc proposed by ACR/EULAR in 2013 were met and the diagnosis of ILD had been made with HRCT [10]. Data for selected demographic and clinical characteristics including age, sex, disease duration (time from the onset of first non-Raynaud symptom attributable to SSc), survival status, disease subtype (limited/diffuse), organ manifestations, autoantibody specifity, and treatment details were collected. Pulmonary arterial hypertension (PAH) was defined as resting mean pulmonary arterial pressure (mPAP) ≥ 25 mmHg and pulmonary capillary wedge pressure (PCWP) < 15 mmHg by right heart catheterisation in the absence of other causes of precapillary pulmonary hypertension [11]. Data for forced vital capacity (FVC) was collected if performed within 12 months of HRCT and expressed as a percentage of predicted values.

The first available HRCT scan of each patient was separately evaluated by two radiologists with 2-year and over 30-year experience in thorax radiology, blinded to clinical information. ‘Definite PPFE’ was defined as upper-lobe predominant pleural thickening with associated subpleural fibrosis with minimal or no involvement of lower lobes (Figure a). Radiologic lesion ‘consistent with PPFE’ was used for the cases where upper lobe pleural thickening with associated subpleural fibrosis was observed but 1) not mainly located in the upper lobes or 2) with features of coexistent disease elsewhere (Figure b) [12]. Additionally, cases with extension to the apical region with a pattern similar to the basal involvement were also included in this group (consistent with PPFE). Patients with ‘definite’ and ‘consistent with’ PPFE lesions were both included in the PPFE (+) group. Patients with apical lesions which are considered secondary to another pathology such as pneumoconiosis and “patients with apical cap” were not included in the PPFE (+) group. ‘Apical cap’ was defined as irregular subpleural opacities smaller than 5 mm diameter and located in the apical region (Figure c). The radiologic pattern of ILD was also classified according to the American Thoracic Society/European Respiratory Society Statement (2013) and Fleischner society guidelines (2018) [6,13]. Patients with NSIP pattern were further classified as having a cellular or fibrotic pattern. Traction bronchiectasis, lung volume loss, axial interstitial thickening and coarse reticular opacities were considered as imaging features of fibrotic NSIP. Cellular NSIP was defined as the presence of only fine reticular opacities and/or ground glass opacities without any of the fibrotic NSIP features [14]. The extent of ILD on HRCT was classified as ‘limited’ or ‘extensive’ according to the staging system described by Goh et al [15]. Emphysema on HRCT was defined as subpleural well-defined cystic areas (paraseptal) and/or low-attenuation areas smaller than 1 cm located in the center of the secondary pulmonary lobule (centrilobular) [16]. Two separate measurements for the proximal 2/3 of the esophagus were made superior and inferior to the aortic arch in axial plane of HRCT scans, respectively. The largest distances between the internal esophageal mucosal borders were measured in the soft tissue window. A distance of >10 mm was considered as esophageal dilatation [17]. Disagreements between radiologists were resolved by consensus.

### 2.1. Statistical analysis

Statistical analysis was performed using SPSS Statistics for Windows version 21.0. Categorical variables were expressed as a percentage. Continuous variables were expressed as mean (standard deviation, SD) for normally distributed data or median (interquartile range, IQR) for skewed data. Normality of data was assessed using Kolmogorov-Smirnov test. Chi-square and Fisher’s exact tests were used to compare categorical variables. Student’s-t test or Mann Whitney-U test were used to compare continuous variables between independent groups. A significance level of 0.05 was used for all statistical tests.

## 3. Results

Among 284 patients with SSc, 152 (53.5 %) had ILD and 105 (93 females) had available HRCT scans. Mean age at disease onset and first available HRCT were 40.8 (13.0) and 49.5 (12.4) years, respectively. The median disease duration was 6.8 (2.0–13.6) years. Thirty- three (32.3%) patients had diffuse disease and 9 (8.8%) patients had overlapping myositis. One (0.9%) patient had scleroderma renal crisis. Most of the patients (67.6%) had positive serology for antitopoisomerase antibodies.

The radiologic pattern of ILD on HRCT was consistent with NSIP in 95 (90.4%), UIP in 9 (8.5%) and bronchiolitis obliterans organizing pneumoniae (BOOP) in 1 patient. Among patients with NSIP pattern, 81 (85.3%) had fibrotic and 14 (14.7%) had cellular NSIP. Apical lesions on HRCT were present in 38 (36.2%) patients. After excluding 18 patients with apical cap and 1 patient with pneumoconiosis (silicosis), PPFE-like lesions on HRCT were present in 19 (18.1%) patients (‘definite PPFE’ in 13 and ‘consistent with PPFE’ in 6 patients). Demographic and clinical characteristics of patients by PPFE-like lesion status are summarized in Table. Compared to PPFE (−) ones, patients with PPFE were older and had a longer disease duration (p < 0.05 for both). Radiologic UIP pattern was more frequent in patients with PPFE (26.3% vs. 4.7%, p = 0.01). PPFE was present in 17.3% of patients with fibrotic NSIP and none of the patients with cellular NSIP (p = 0.12). The median time period between HRCT and FVC measurement was 0.7 (0.06–3.16) months. Patients with PPFE had lower median FVC than those without (64% vs. 82%, p = 0.005). Although follow-up duration after the first available HRCT was shorter, more deaths occurred in the PPFE (+) patients during this period (31% vs. 11%, p = 0.04). Causes of deaths were as follows: in PPFE (+) group; PAH in 1, pneumoniae in 1, gastrointestinal haemorrhage and sepsis in 1, lung cancer in 1, unknown in 2 patients, in PPFE (−) group; PAH in 2, lung cancer in 1, acute leukemia in 1, sepsis due to osteomyelitis in 1, pneumoniae in 1, heart failure in 1, pneumoniae/exacerbation of ILD in 1, scleroderma renal crisis in 1 and unknown in 1 patient. Myositis overlap was slightly more frequent in patients with PPFE (15.8% vs. 7.0%, p = 0.20). Spontaneous pneumothorax occurred in 1 patient with definite PPFE during follow-up. Fourteen patients with PPFE had consecutive HRCT scans. During a median time period of 3.5 (2.4–6.3) years, 5 patients had progression of PPFE. The median time period between the index and follow-up HRCT scan was higher in patients with progression of PPFE than stabile ones (6 (4–13.5) vs. 3 (2–4.2) years, p = 0.09).

## 4. Discussion

The aim of this study was to present data about the prevalence and clinical associations of radiologic PPFE in a single-center cohort of SSc-ILD patients. The prevalence of PPFE was found as 18.1%. Patients with PPFE more frequently had radiological UIP patterns and lower FVC values than PPFE (−) ones.

The prevalence of radiologic PPFE in our cohort is in line with a recent study analyzing SSc-ILD cohorts of two referral centers from Italy and UK. The frequency of PPFE was found 18.4% in the UK cohort and %17.5 in the Italian cohort [8]. Another study reported a higher frequency of radiologic PPFE-like lesions in patients with SSc-ILD (6/14, %43) however, their definition did not exclude apical cap which is a relatively common finding in HRCT [18].

Patients with PPFE had more severe restrictive lung disease in our study. Longer disease duration and higher age of PPFE (+) patients are potential explanations for this finding. On the other hand, in a previous study on SSc-ILD, Bonifazi et al. found that patients with PPFE had trend towards lower FVC values (74 vs. 80, p = 0.1) and higher annual decline in FVC compared to PPFE (−) patients despite comparable disease duration and average ILD extent on HRCT [8]. Similarly, Enomoto et al. reported lower FVC values in PPFE (+) patients at the time of ILD diagnosis in a connective tissue disease associated ILD (CTD-ILD) cohort (67% vs. 77%, p: 0.15) [18]. Previous studies showed that both lower and upper lobe involvement more frequently occurs in PPFE (+) patients with CTD-ILD [18]. Reticular abnormalities and ground glass opacities are also known to be more intense in these patients compared to PPFE (−) ones [18]. Similar to previous studies, radiologic UIP patterns were more frequent in PPFE (+) patients in our cohort. In addition, patients with extensive (>20%) lung fibrosis were more frequent in PPFE (+) group and PPFE was not observed in any patients with cellular NSIP. PPFE with a lower lobe predominant UIP pattern is a previously known entity [12,19,20] and shown to be more frequent in secondary PPFE than primary PPFE [19]. Radiologic PPFE-like appearance may be a result of apical involvement by UIP in these patients. In a pathological examination, Enomoto et al. showed upper lobe UIP in 2 of 3 patients with radiologically PPFE-like lesions. Taken together, lower FVC in PPFE (+) patients in our study may be explained by more extensive involvement of lungs by UIP.

The negative impact of PPFE on the prognosis of interstitial pneumoniaes such as idiopathic pulmonary fibrosis (IPF) has been previously defined [19]. Bonifazi et al. reported that the presence of radiologic PPFE was also associated with poorer prognosis in SSc-ILD. Similarly, Enomoto et al. showed that presence of PPFE is an independent predictor of death due to respiratory causes in patients with CTD-ILD. The mechanism of the impact of PPFE on prognosis in CTD-ILD is unclear. Besides a possible direct effect on prognosis, it may simply be an indicator of extensive lung fibrosis. Although our data did not allow us to perform survival analysis, more deaths occurred in PPFE (+) patients despite a shorter follow-up duration. However, longer disease duration and higher age in PPFE (+) group likely might have biased our results. Nevertheless, the presence of PPFE may be of value in prognostic evaluation of patients with SSC-ILD, as it can be easily assessed on HRCT in daily practice.

Retrospective single-center design and a limited number of patients are major limitations of this study. Second, we could not provide information about the infections which are one of the most common etiologies of PPFE. Lastly, longer disease duration at first HRCT did not allow us to draw any conclusion about prognostic value of PPFE in Ssc-ILD.

To conclude, PPFE on HRCT is not an uncommon lesion in SSc-ILD and associated with radiologic UIP pattern, more extensive lung involvement, and worse lung functions. Further studies including patients with shorter disease duration are needed to elucidate the prognostic value of PPFE in SSc-ILD.

## Figures and Tables

**Figure a,b,c:**
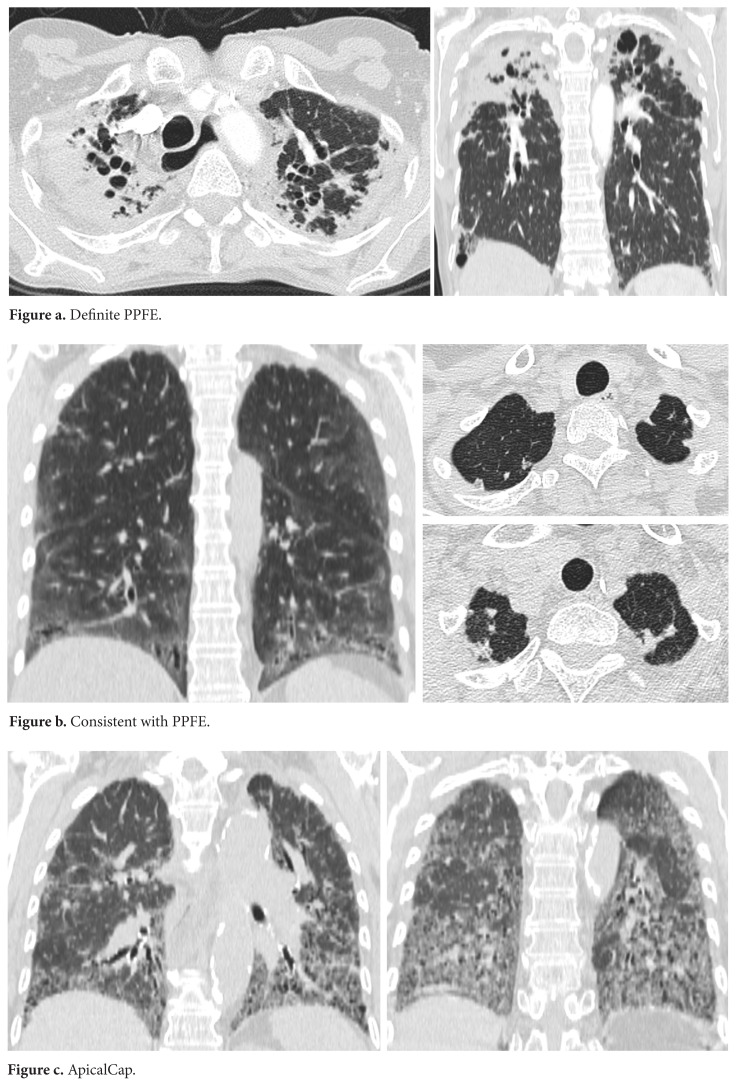
High resolution thorax tomography images of pleuroparenchymal fibroleastosis like lesions and apical cap

**Table:** Demographic and clinical characteristics of patients.

	All patients (N = 105)	PPFE (−) (N = 86)	PPFE (+) (N = 19)	P

**Female**	93 (88.5)	77 (89.5)	16 (84.2)	0.45

**Age, years**	49.5 (12.4)	47.9 (12.2)	56.6 (11.3)	**0.005** a

**Disease duration, years, median (Q1–Q3)**	6.8 (2.0–13.6)	5.6 (1.4–11.2)	15.7 (6.6–18.9)	**0.002** b

**Follow-up duration, years, median (Q1–Q3)**	6.2 (2.5–8.5)	6.6 (3.3–8.8)	2.3 (1.5–7.4)	**0.017** b

**Diffuse/limited disease**	33/72	27/59	6/13	0.98

**Antinuclear antibody (+)**	100 (95.2)	82 (95.3)	17 (89.4)	1.000

**Antitopoisomerase (+)**	71 (67.6)	59 (68.6)	12 (63.1)	0.87

**Anticentromere (+)**	3 (2.8)	2 (2.3)	1 (5.2)	0.43

**Digital ulcers (active or previous)**	62 (60.2)	50 (59.5)	12 (63.2)	0.77

**Myositis**	9 (8.5)	6 (7.0)	3 (15.8)	0.20

**Pulmonary arterial hypertension**	9 (8.5)	6 (7.0)	3(15.8)	0.20

**Usual interstitial pneumoniae**	9 (8.5)	4 (4.7)	5 (26.3)	**0.01**

**The extent of ILD on HRCT**				
**Limited (<20%)**	57 (54.3)	52 (60.5)	5 (26.3)	**0.01**
**Extensive (>20 %)**	48 (45.7)	34 (30.5)	14 (73.7)	

**Emphysema on HRCT**	10 (9.5)	9 (10.5)	1 (5.3)	0.68

**Esophageal dilatation on HRCT**	97 (92.4)	79 (91.9)	18 (94.7)	1.000

**Forced vital capacity %, median (Q1–Q3)** *	81 (67–92)	82 (72–93)	64 (52–83)	**0.005** b

**Treatment**				
**Cyclophosphamide**	64 (60.9)	50 (58.1)	14 (73.7)	0.20
**Mycophenolatemofetil**	43 (40.9)	34 (39.5)	9 (47.4)	0.53
**Rituximab**	13 (12.3)	11(12.8)	2 (10.5)	1.000
**Vasodilators (ERA, PDE5-i, intravenous prostanoids)**	42 (40.0)	33 (38.4)	9 (47.4)	0.47

ILD; interstitial lung disease, HRCT; high resolution computed tomography, ERA; endothelin receptor antagonists, PDE5-i: phosphodiesterase 5 inhibitors.

Values are n (%) and mean (SD) unless otherwise specified.

a Student’s t-test was used.

b Mann-Whitney U test was used.

* FVC measurements were available in 73 and 13 patients with and without PPFE, respectively.
